# A systematic review and meta‐analysis investigating the impact of childhood adversities on the mental health of LGBT+ youth

**DOI:** 10.1002/jcv2.12079

**Published:** 2022-05-10

**Authors:** Lucy Jonas, Gonzalo Salazar de Pablo, Mamie Shum, Chiara Nosarti, Chris Abbott, Julio Vaquerizo‐Serrano

**Affiliations:** ^1^ Institute of Psychiatry Psychology and Neuroscience King's College London London UK; ^2^ South London & Maudsley NHS Trust London UK; ^3^ Institute of Psychiatry, Psychology and Neuroscience King's College London West London NHS Trust London UK

**Keywords:** adverse childhood experiences, LGBT+ youth, mental health outcomes, meta‐analysis

## Abstract

**Background:**

The presence of trauma as a backdrop to the lives of LGBT+ youth has been recognised in recent literature. LGBT+ youth report a higher frequency, severity and pervasiveness of adverse childhood experiences when compared to their heterosexual and cisgender counterparts. This exposure has been directly related to an increased risk of mental health problems.

**Method:**

A systematic literature search of Medline, Embase, PsycINFO, PubMed and Web of Science was conducted from the date of their inception until the 1st September 2021. The study protocol was registered in PROSPERO (CRD42021240472).

**Results:**

A total of 27 studies satisfied the inclusion criteria and were used in the systematic review, representing 199,285 participants, 26,505 of whom identified as LGBT+ (mean age 16.54). Female participants (ranging from 11% to 74%) and white participants (7.7%–96%) made up the largest percentage of most samples. Depressive symptoms were the most commonly described psychiatric outcome (*n* = 17, 63%), followed by anxiety symptoms (*n* = 6, 31.5%). 18 studies provided meta‐analysable data, compromising 21,781 LGBT+ young people. LGBT+ youth reported a higher prevalence of adverse experiences in comparison to their heterosexual or cisgender counterparts (*p* < .001), with sexual abuse representing the most commonly reported experience (29.7%), followed by verbal abuse (28.7%), physical abuse (26.5%) and cyberbullying (19.1%). LGBT+ youth were also at a heightened risk of mental health disorders (*p* < .001), with 36.9% and 31.5% of sample meeting the clinical criteria for depression and anxiety, respectively.

**Conclusions:**

Continued advocacy is needed from communities and Allies to support and empower LGBT+ youth in the face of adversity. Longitudinal and longer‐term studies are required to further understand the relationship between adverse experiences in LGBT+ youth and the impact on mental health.

**Key points**

- LGBT+ youth report a higher prevalence of adverse experiences in comparison to their heterosexual or cisgender counterparts (*p *< .001), with sexual abuse the most commonly reported
- LGBT+ youth were at a heightened risk of mental health disorders (*p* < .001), evidenced by elevated rates of depression and anxiety in LGBT+ youth (36.9% and 31.5%, respectively)
- Continued advocacy is needed from communities and allies to support and empower LGBT+ youth in the face of adversity

## INTRODUCTION

The first study to assess adverse childhood experiences (ACEs) captured a series of ongoing stressful or traumatic events experienced by an individual before the age of 18 (Felitti et al., 1998). The most commonly reported included physical, psychological and sexual abuse, physical and emotional neglect, and five aspects of household dysfunction. Previous research suggests that the risk of exposure to a traumatic event escalates for individuals with minority identities related to sexual orientation, gender expression, or gender identity (the LGBT+ community; Clements‐Nolle et al., 2018). They are the target of unwanted victimisation across a wide range of contexts, for example, parental physical assault, harassment at school, and on social media platforms (Williams et al., 2021).

Despite burgeoning global activism and statutory advances in legislative reform, research continues to document how sexual and gender minority groups are at greater risk of mental health difficulties (Semlyen et al., 2016). This is illustrated by increased prevalence of depressive symptoms, anxiety, substance abuse disorders, eating disorders and behavioural disorders (Austin et al., 2013; Day et al., 2017; Russel & Fish, 2016; Scannapieco et al., 2018). The most frequently invoked explanation for this stems in part from a disproportionate exposure to traumatic events (Blosnich & Andersen, 2015). It is suggested that adversity exposure in childhood can impact healthy emotional growth (Spinazzola et al., 2005). For example, in LGBT+ adults, adversity has been linked with depressive symptoms, anxiety, isolation, rejection, and eating and behaviour disorders (Blosnich & Andersen, 2015).

### Importance

We know thus far that LGBT+ individuals face a significant and poorly understood set of ACEs and bear a higher burden of mental health disorders than the general cohort. However, previous research has focused more on outcomes in adulthood rather than adolescence (Blosnich & Andersen, 2015).

### Study objectives

The current review aims to be the first to present findings of studies measuring associations between adverse experiences and mental health outcomes across the full spectrum of LGBT+ youth. We aim to fill the gaps in existing research by (1) examining the prevalence rates of trauma in the lives of LGBT youth, (2) synthesising the mental health implications of ACEs, and (3) comparing the presence of mental health disorders in individuals exposed to ACEs compared to those not exposed.

## METHODS

This study was conducted in accordance with the Preferred Reporting Items for Systematic Reviews and Meta‐analyses Reporting Guideline (Page et al., 2021) and the Meta‐analysis of Observational Studies in Epidemiology (MOOSE) reporting guidelines (Stroup et al., 2000; Tables S1 and S2). The study protocol was registered in PROSPERO (CRD42021240472).

### Information sources

A multistep search of the literature was undertaken by two independent researchers (LJ and JV) through the Web of Science database (Clarivate Analytics), incorporating the Web of Science Core Collection, BIOSIS Citation Index, KCI‐Korean Journal Database, MEDLINE, Russian Science Citation Index, SciELO Citation Index, Cochrane Central Register of Reviews and Ovid/PsychINFO databases from inception until 1st September 2021. We also searched for unpublished data on OpenGrey European database, MedRxiv and PsyArXiv to prevent publication bias. The search was completed reviewing sections of articles to identify any relevant citations. Based on an initial scoping search, we searched Medical Subjects Headings (MeSH) and keywords related to: (1) LGBT+ youth, (2) childhood adversity, and (3) psychiatric related health conditions (delineated in Table S3).

### Eligibility criteria

Included studies were those that (i) studied young people with a mean age of ≤18 identifying as LGBT+; (ii) examined the relationship between childhood adversity and mental health outcomes in LBGT+ individuals; (ii) included exposure to trauma subtypes; (iii) reported outcomes compatible with a diagnosis from the *Diagnostic and Statistical Manual of Mental Disorders* (DSM) or *International Classification of Diseases* (ICD); (iv) provided relevant data on the relationship between any form of adversity and mental health; (v) were empirical, longitudinal or transversal, with original data, and (vi) had reports written in English. Consideration of the amount of available research led to the inclusion of all countries, settings, and developmental ages.

Studies with insufficient information were included in the review but excluded from the meta‐analysis (Lipsey & Wilson, 2001). Additional inclusion criteria for the meta‐analysis were those with (i) meta‐analysable data and (ii) non‐overlapping samples. Overlap between the included studies was actively searched by evaluating the country, recruitment period, and setting from which the study sample was obtained.

### Data extraction and outcome measures

Independent data extraction was performed by two researchers (LJ and MS), and discrepancies were resolved through discussions with the senior author (JV). If available, the variables extracted included: study and sample‐level descriptors (first author and year; study name; country; recruitment; design; sample size; age: mean [SD], % females; population category; sexuality measure); ACE information (exposure category, instrument to assess the traumatic experiences and the proportion in the ACE group); diagnoses/outcomes information (mental health disorder and diagnostic instrument) and all relationships of interest were reported along within the main and quantitative findings.

### Quality assessment

Study quality was evaluated in all the included studies. The Newcastle‐Ottawa scale (NOS; Wells et al., 2000) was performed to avoid incorporating any methodologically deficient or biased literature. Variations of the scale (Alameda et al., 2020; Modesti et al., 2016) were needed to assess different study designs (Table S4). Decisions specific to the paper were made by the authors about representative cohorts, important factors to control for and adequate response rates.

### Data synthesis and meta‐analysis

As the studies in this meta‐analysis were expected to be heterogeneous, the random‐effects model was used (DerSimonian & Laird, 1986). Heterogeneity between studies was measured with the Q statistic and its magnitude was evaluated with the I‐squared index (Lipsey & Wilson, 2001). A sensitivity analysis was performed stratified by group, that evaluated the design of the study to determine whether there were differences between cross‐sectional and longitudinal studies. Publication bias was assessed by visually inspecting funnel plots (Sterne et al., 2001) and applying the regression intercept of Egger (Egger et al., 1997). Finally, meta‐analytical regressions were conducted to evaluate the association between our outcomes and the factors that might be associated with the presence of any ACE or the presence of a mental health problem in LBGT+ youth. The fixed meta‐regressor factors were age, gender (female) and ethnicity (white). All *p*‐values reported in the meta‐analysis were two‐sided and the level of significance was set at a *p*‐value of less than .05. Comprehensive Meta‐analysis Software, version 3 (Biostat, Inc; 26) was used (Borenstein et al., 2005).

## RESULTS

### Screening and selection

**FIGURE 1:**
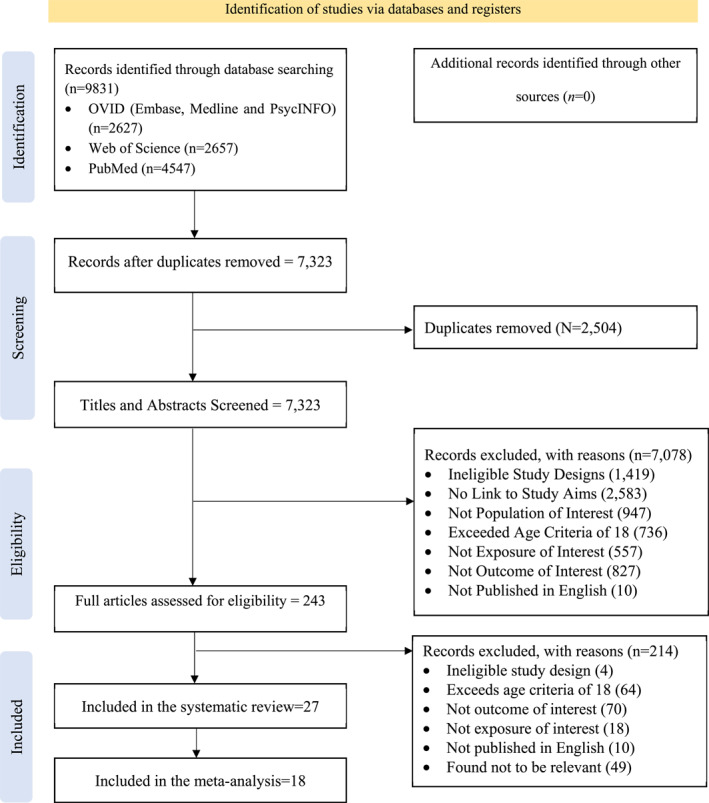
PRISMA 2020 flow diagram for new systematic reviews (Page et al., 2021)

The search strategy was initially broad with 9831 articles retrieved through the initial database search. Following the removal of duplicates (*n* = 2504), 7323 titles and abstracts were screened. 7078 studies were inconsistent to the inclusion criteria and these efforts produced a list of 243 unique citations. Of this set of articles, a further 214 were removed, leaving 27 studies (Table S5). Three independent reviewers randomly selected and reviewed the texts to ensure eligibility. After removing studies that did not provide meta‐analysable data, 18 studies were included in the meta‐analysis (Figure 1; PRISMA 2020 flow diagram).

### Study characteristics

This review includes 199,285 participants, of whom 26,505 identified as LGBT+. The range of LGBT+ participants ranged between 8.3% and 100% across the included studies. Seven studies incorporated all LGBT+ subpopulations, 17 studies used just sexual minority populations, one focused solely on bisexual youth (Pollit et al., 2017) and two focused on transgender/non‐conforming youth (Chozden et al., 2019; Veale et al., 2017). Of the studies that provided a breakdown of sexual identities (*n* = 25), the category of both sex attracted made up the largest range across studies (2.2%–100%) followed by same sex attracted (1.3%–49%; referenced in Table S6).

#### Sociodemographic data

The majority of studies (*n* = 22) reported on the mean age of participants. All mean ages were 18 or below (mean age = 16.45), with a small proportion of participants exceeding 18 but not skewing the mean. The ethnic makeup of the samples varied greatly from study to study. White participants made up the largest range across studies (7.7%–96%). In 6 studies, racial/ethnic participants represented at least 50% of the sample, with a range between 4% and 72%. Female participants made up the largest percentage of most studies (*n* = 16), ranging between 11% and 74% (referenced in Table S7).

#### Geographical information

Eigteen studies (66.67%) were from the USA, but the set also included studies from Asia (*n* = 2, 7.4%), Canada (*n* = 1, 3.7%) and Europe (*n* = 6, 4.5%; referenced in Table S6).

#### Study design

The majority of the studies were cross sectional (*n* = 23, 85.2%), with four (14.8%) longitudinal studies (Burton et al., 2013; Hatchel et al., 2018; Jones et al., 2017; Mittleman, 2019).

#### Quality assessment

The quality of the studies ranged from 5 to 9 using the Newcastle‐Ottawa scale (NOS). The full results are detailed in Tables S8 and S9.

#### Exposures of interests

A range of adverse experiences were examined (Table 1). A range of 7.04% (Kurki‐Kangas et al., 2019) to 99.92% (Zhao et al., 2020) of LGBT+ youth were reported to have experienced an ACE. Studies measuring victimisation as a distinct category, such as combining being teased or bullied, hit or beaten up, treated unfairly and public ridicule due to actual or perceived sexual minority status, had a range of 4% (Lowery et al., 2020) to 69% (Veale et al., 2017). Six studies examined sexual abuse (Atteberry et al., 2020; De Laney et al., 2020; Donahue et al., 2017; D’Augelli et al., 2006; Hatchel et al., 2018; Zhao et al., 2020). Nine percent (D’Augelli et al., 2006) to 48% (Hatchel et al., 2018) of LGBT+ youth samples reported sexual assault and unwanted or uncomfortable sexual experiences (e.g., rape, attempted rape or made to perform sexual acts). Eleven percent (D’Augelli et al., 2006) to 69% (Veale et al., 2017) reported physical abuse (*n* = 7 studies; Button et al., 2016; D’Augelli et al., 2006; Donahue et al., 2017; Huebner et al., 2015; Lowry et al., 2020; Veale et al., 2017; Zhao et al., 2020) and 11% (Lowry et al., 2020) to 78% (D’Augelli et al., 2006) reported verbal abuse (*n* = 7; Button et al., 2016; D’Augelli et al., 2006; Donahue et al., 2017; Huebner et al., 2015; Lowry et al., 2020; Veale et al., 2017; Zhao et al., 2020). Three studies focused on cyberbullying and the power imbalance between perpetrator and victim on a virtual platform (Byrd, 2015; Garaigordobil & Larrain, 2020; Duarte et al., 2018), ranging from 11.76% (Zhao et al., 2020) to 47% (Byrd, 2015). Three studies examined unique LGBT+ variables. One study (Pollit et al., 2017) examined the impact of disclosure stress to friends and family, another measured the degree of comfort regarding a transgender person's identity and appearance (appearance congruence; Chozden et al., 2019). Lastly, one study (Veale et al., 2017) used a stigma index based on reports of discriminatory behaviours.

**TABLE 1:** Exposures of interests and outcomes measures

First author	Exposure category	Exposure measure	Diagnosis/symptoms	Instrument	Proportion in Ace group
Attebery Ash	Bullying; sexual violence	HKCS	MDD	One item pertaining to sadness	321 (50.9%) experienced sexual violence.
Baams	Victimisation	SOVS	MDD	6‐Item depressive mood list	Male M (SD): 1.40 (.38) female M (SD): 1.33 (.43) experienced victimisation.
Battalen	Childhood trauma; Discrimination	CTQ, EDS, IND	MDD, suicide ideation, suicide attempts	CES‐D, C‐SSRS	64% (*n* = 62) reported being victimized due to their gender identity or sexual orientation.
Birkett	Bullying victimisation; homophobic teasing	Illinois aggression scale	MDD, suicidality	Single item question (and suicidality question)	*M* = .64 (SD = .98). For LGB, *M* = 1.29(1.35) for questioning
Burton	Victimisation	Victimisation due to actual or perceived sexual minority status assessed via four items.	MDD; suicidality	CES‐D; *“Have you ever thought about or attempted to kill yourself?”*	52% reported being victimised.
Button	Victimisation	Victimisation scale	Substance use disorder mental health problems	Suicidality scale; grades in the last 12 months	52.6% reported victimisation and 46.2% report considering suicide.
Byrd	Cyberbullying	CVS	MDD; GAD	CES‐D; SCARED	91.4% of SGM youth reported cyberbullying victimisation.
Chodzen	Gender identity AC; Internalized transphobia	AC subscale of the TCS	MDD; GAD	Y1‐4	*n.r.*
D’Augelli	SOV; verbal, sexual or physical.	Assessed using questions from other research papers (D'Augelli, 2002)	Overall mental health, PTSD	BSI, GSI, TSC, DISC	78% reported verbal SOV, 11% reported physical SOV, and 9% reported sexual SOV.
DeLaney	Sexual victimisation	LEC	Depression, PTSD, alcohol use disorder	SCL‐90; PC‐PTSD16‐item measure from AUD criterion (DSM‐V)	Sexually victimized LGBQA students: Depression (24%), PTSD (45%); AUD symptoms 35%
Donahue	Victimisation experience	Dichotomous variable based on reports of experiencing emotional abuse, physical abuse/neglect, sexual abuse/assault	ADHD, eating disorders, AUD, MDD, Drug use disorder, anxiety,	Scared; CES‐ D; ASRS‐v1.1; EDI; AUDIT; DUDIT	Any type of victimisation was reported by *n* = 176 (53.2%) of LGBT+ participants.
Duarte	Cyberbullying	Two relevant items from the student school survey	Depression; PTSD	PHQ‐9; CPSS	32/74 (43.2%) LGB participants reported cyberbullying
Garaigordibil	Cyberbullying	Screening of peer harassment (physical, verbal, social and psychological)	Depression; anxiety	BDI‐II; SAS‐A	25.1% (*n* = 55/218.5) were victims of severe bullying.
Hatchel	Sexual harassment; violence victimisation	A reduced 6 item version of AAUWSHS	Depression	8‐Item version of MDS	In wave 3, the mean of sexual harassment victimisation was 1.61 (SD = .74)
Huebner	School victimisation	Nine questions that assessed the frequency with which a variety of events had occurred	Substance abuse	Measures used in the NLSAH	67.1% SMY reported physical fights (*n* = 338); 48% sexual jokes (*n* = 242)
Jones	Bullying	At 16 years, a questionnaire asked participants to report if they had experienced ‘bullying by another person ’since the age of 12 years.	Anxiety	ICD‐10 diagnosis of any anxiety disorder according to the computerised CIS‐R.	*N* = 110 SMY reported bullying
Kurki‐Kangas	Bullying	2 questions derived from a world health Organization study on youth health	Depression; anxiety	Depression was measured with 2 screening questions. GAD‐7	Males (13.7%, ‐ 27.8%); females (3.3%–11%) reported bullying.
Li	Adverse childhood experiences	BRFSS	NSSI	CDC YRBS	Sexual (*n* = 56) + physical (130) abuse; household violence (510), parental mental illness (1588) and substance abuse (102).
Lowry	Violence victimisation	5 categories were assessed to create a 3‐level count variable.	Cigarette, alcohol and marijuana use	YBRS	Up to 12.8% of males reported victimisation and 17.7% females.
McNamee	Bullying	Closed questions relating to bullying in their school.	Mental health	GHQ	47.1% of LGB respondents reported being bullied.
Mereish	Homophobic bullying	They were asked how often they were bullied during the past 12 months “because you are gay, lesbian, or bisexual, or someone thought you were.”	Substance use	Questions pertaining to alcohol use, illicit drug use, prescription drug use and cigarette consumption in their lifetime (lifetime and recent)	*n. r*
Mittleman	Peer victimisation	Parents were asked whether the child “gets teased”. Children were asked about peer victimisation.	Mental health	CBCL/6–18; CES; BSI	*T* = 13.4%, *T*2 = 68%, *T*3 = 40%.
Peters	Abuse; peer victimisation	4 item peer victimisation scale; parent reports and medical charts.	Depression; NSSI	BDI‐II; C‐SSRS	19/27 SMA reported abuse (70%).
Pollit	Disclosure stress	Items from the LGBTQ Coming out stress scale	Depression	BDI for youth	Stress of disclosure to family (male: 1.60, 1.3; female: 2.05, 1.3); to friends (0,90, 1.2 and female 1.51, 1.3)
Price‐Feeney	Bias victimisation	All participants were queried about whether other youth had aggressed on them for specific reasons.	Depression	CES‐D	50% of youth reported that they experienced at least one of the seven forms of bias‐based victimisation.
Veale	Enacted stigma	Enacted stigma index on discriminatory behaviours (e.g., verbal harassment, bullying, abuse or exploitation).	NSSI, suicide	‘‘During the past 12 months, how many times did you attempt suicide?’’; GWS.	63% had experienced some form of sexual orientation harassment and 69% reported gender identity harassment.
Zhao	Childhood maltreatment/bullying victimisation	CTQ‐SF, OBQ	DepressionAnxiety	CES‐DGAD‐7 scale	13.1% experienced physical abuse, 24.5% emotional abuse, 19.6% sexual abuse, 23% bullying, 18% cyberbullying.

#### Outcomes measures

Various mental health outcomes were studied, and diverse measures were used to assess the same constructs across studies (Table 1). The prevalence of LGBT+ youth meeting criteria for an identifiable mental health problem ranged from 8.3% (Donahue et al., 2017) to 82% (Battalen et al., 2020) and were most common in LGBT+ youth who had experienced ACEs.

From the available data, the most commonly described psychiatric outcome was depressive symptoms (*n* = 17, 63%), which ranged between 12% (Peters et al., 2020) to 82% (Byrd, 2015) of LGBT+ adolescents. Anxiety symptoms was the focus of six studies (22.2%; Byrd, 2015; Chodzen et al., 2019; Donahue et al., 2017; Garaigordibil et al., 2020; Jones et al., 2017; Kurki‐Kangas et al., 2019; Zhao et al., 2020), and ranged between 18.3% (Kurki‐Kangas et al., 2019) and 72% (Byrd et al., 2015) of LGBT+ adolescents. Furthermore, three studies examined the impact of trauma on nonsuicidal self‐injury or NSSI (Li et al., 2019; Peters et al., 2020; Veale et al., 2017). Of note, for each discriminatory behaviour experienced, participants were 25% more likely to have reported NSSI in the past year (Veale et al., 2017). Three studies reported PTSD symptoms as a result of experiencing ACEs, where 9% (D’Augelli et al., 2006) to 33.9% (Duarte et al., 2018) met the criteria for a diagnosis. Sexual victimisation explained 23.3% of variance in PTSD disorder (DeLaney et al., 2020). Six studies looked at alcohol use, abuse or dependence. Four studies pertained to excessive alcohol use (Birkett et al., 2009; Button et al., 2016; Huebner et al., 2015; Meriesh et al., 2017), with 1.4% of one sample binge drinking almost daily (Huebner et al., 2015). Two studies reported significant correlations between ACEs and alcohol use disorder (DeLaney et al., 2020; Donahue et al., 2017). Lastly, one of the included studies reported direct correlations between exposure to victimisation and eating disorder symptoms, ADHD and autism spectrum disorder (Donahue et al., 2017).

### Results of the meta‐analysis

Eighteen studies had data that allowed meta‐analysis, comprising 21,781 LGBT+ individuals (Attebery et al., 2020; Battalen et al., 2020; Button, 2016; Byrd, 2015; Donahue et al., 2017; Duarte et al., 2018; Garaigordobil & Larrain, 2020; Jones et al., 2017; Kurki‐Kangas et al., 2019; Li et al., 2019; Lowry et al., 2020.; McNamee et al., 2008; Mittleman, 2019; Peters et al., 2020; Price Feeney et al., 2018.; Veale et al., 2017; Zhao et al., 2020).

#### Proportion of LGBT+ youth exposed to any adverse childhood Experience

**FIGURE 2:**
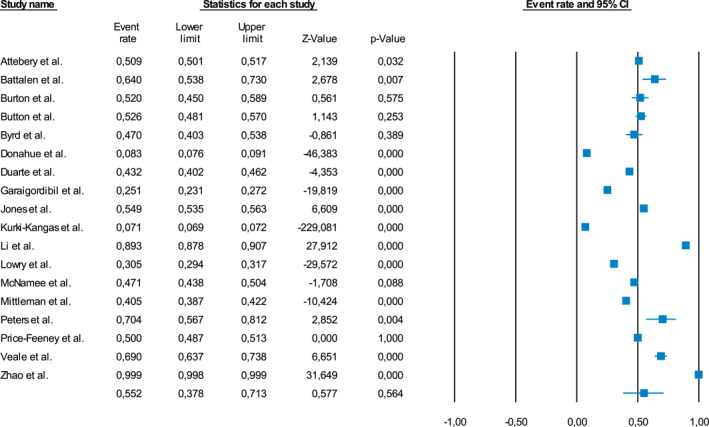
Proportion of LGBTQ+ youth exposed to any adverse childhood experience. Forest plot

Overall, the meta‐analytical results revealed that 55.2% (95%CI = 37.8–71.3%, *k* = 18, *n* = 186,468) of LGBT+ youth were exposed to any adverse experience. The meta‐analysis on the type of Adverse Childhood Experiences (ACEs) in LGBT+ youth showed that the majority of young individuals had experienced sexual abuse (29.7%, 95%CI = 21.5–39.4%, *k* = 6); followed by verbal abuse (28.7%, 95%CI = 18.3%–41.8%, *k* = 7); physical abuse (26.5%, 95%CI = 17.6–37.7%, *k* = 7), and cyberbullying (19.1%, 95%CI = 10.4–32.5%, *k* = 4). Heterogeneity across the studies included was statistically significant (I2: 00.582–99.984, all *p* < .001) in all the primary analyses (Figure 2, Figure 1).

#### Proportion of LGBT+ youth with any mental disorder

**FIGURE 3:**
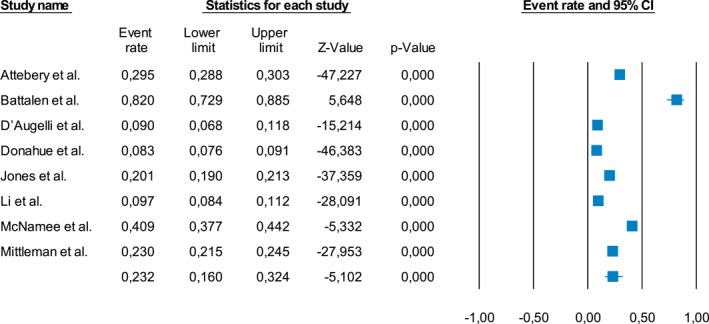
Proportion of LGBT+ youth with any mental disorder. Forest plot

The meta‐analysis on the prevalence of mental health disorders in LGBT+ youth showed that 23.2% had any mental disorder (95%CI = 16.0–32.4%, *k* = 8). Depressive symptoms and disorders were the most prevalent in the sample (36.9%, 95%CI = 26.1–49.1%, *k* = 5), followed by anxiety disorders (31.5%, 95%CI = 25.6–38.2%, *k* = 5). Heterogeneity across the studies included reporting mental health conditions within a sample of LGBT+ individuals was statistically significant (*I* ^2^: 99.456–99.849, *p* < .001) (Figure 3, Figure 2).

Furthermore, all our outcomes in terms of mental health conditions were more prevalent and statistically significant in LGBT+ individuals compared to heterosexual youth (all *p* < .001). The sensitivity analyses comparing the studies by their design (cross‐sectional vs. longitudinal studies) for both sets of analyses (proportion of LGBT+ youth exposed to any ACEs and proportion of LGBT+ youth with any mental health problem) did not reveal any statistically significant differences. Lastly, meta‐regression analyses, controlled for age, gender and ethnicity, showed that an older age (β = 0.227, *p* = 0.0003) was associated with a higher prevalence of any ACEs in LGBT+ youth. There was no significant association between gender and ethnicity and the presence of any ACEs (all *p* > .05) nor between age, gender or ethnicity, and the presence of mental health conditions (all *p* > .05).

Further information and figures produced by the meta‐analysis are shown in the supporting information.

## DISCUSSION

This is the first meta‐analysis that comprehensively addresses the presence of ACEs in LGBT+ youth. A thorough search of relevant research yielded no existing reviews that investigated a range of adverse experiences across the full spectrum of LGBT+ youth and mental health outcomes. We systematically reviewed 27 studies, focusing on the distinctive features of adversity in childhood and mental health outcomes in 22,309 LGBT+ adolescents. We further addressed via the meta‐analysis the presence of four forms of adverse childhood experiences and two mental health outcomes experienced by 21,781 LGBT+ youth from 18 articles. In most studies, a higher prevalence of female participants was reported compared to males. Possible explanations include an account of sexual prejudice that posits how negative attitudes towards sexual minority individuals are more severe for males than females (Bettinsoli et al., 2019) and that disclosure stress is more prevalent among men than women (Pollit et al., 2017).

After reviewing the literature, we cast a broad, atheoretical net for multiple types of adversity to overcome limitations of previous ACE reviews, recommending more thorough measures (Craig et al., 2020). The results of the systematic review build on knowledge regarding the different patterns of adverse experiences reported among LGBT+ youth. Our meta‐analysis showed that more than 50% of LGBT+ youth suffered any ACE, which is in keeping with prior literature in which ACEs were reported by 50% of young people (Craig et al., 2020). The most commonly cited forms of victimisation were physical, sexual and verbal abuse, where sexual and gender minority youth have increased odds of exposure to sexual abuse, verbal abuse and physical abuse. Such findings are similar to findings in LGBT+ adults (Andersen & Blosnich, 2013).

The results from our meta‐analysis showed that experiences of sexual abuse were the most prevalent ACE in LGBT youth (29.7% of the sample), with one study finding the risk of experiencing sexual abuse for LGBT youth was tenfold compared to heterosexual youth (Atteberry Ash et al., 2020). This prevalence is in keeping with a national LGBT+ survey reporting a prevalence rate of 33% (Bradford et al., 1997). 26.5% of LGBT+ youth in this sample also reported physical abuse. This result was empirically similar to previous literature reporting that between 22.2% and 33% of LGBT+ students were physically harassed due to their sexual orientation or gender expression (Craig et al., 2020; Kann et al., 2018; Kosciw et al., 2016).

There was a noticeably smaller proportion of the sample reporting verbal harassment (28.7%), compared to previous meta‐analysis reviews (55% in Katz‐Wise & Hyde, 2012). A possible explanation could be the relative recency of most of studies included in the meta‐analysis. In the past decade, we have been witnessing to a social climate becoming more supporting of LGBT+ young people and stricter anti‐bullying policies, which may in turn reduce verbal harassment for example, around school institutions. In this meta‐analysis, 19.1% of LGBT+ youth had undergone cyberbullying. Recent national data from the Youth Risk Behaviour Survey (Kann et al., 2018) found a slightly higher prevalence (27%), but this was still higher than heterosexual counterparts.

The psychological sequelae from victimisation have been shown to increase the risk of mental health problems. We assessed outcomes defined by diagnostic symptoms or the criteria of any mental health disorder present in any edition of the DSM or ICD, to prevent overestimating the prevalence of mental health problems among this population.

A meta‐analysis examining the worldwide‐pooled prevalence of mental disorders affecting children and adolescents was 13.4% (Polanczyk et al., 2015). The meta‐analysis demonstrated that 23.2% of the LGBT+ sample experienced mental health problems, highlighting the disparate rate of mental health symptoms compared to the general population. In our sample, depressive symptoms were the most prevalent mental health problem (36.9%). This study found that LGBT+ youth experienced a higher rate of anxiety than the general population of young people, with a prevalence rate of approximately 31.5%. Contributing factors for the higher prevalence may include the unique stressors and uncertainties that LGBT+ youth may endure. Sharing a sexual orientation identity with close friends and family may represent a significant stressor in an LGBT+ adolescent's life, and possible internalised fear of negative reactions or rejection may deter LGBT+ individuals from disclosure. Sexual orientation disclosure is linked with social anxiety disorder, phobia, or PTSD symptomatology. The implications of these findings are discussed in the supporting information.

### Limitations

This review has several limitations which engender caution when interpreting the results. It is possible that the number of LGBT+ participants is an underestimate of the existing LGBT+ youth. Underestimation can stem from existing social factors such as stigma or discriminatory laws. In four studies, participants were sourced through LGBT+ specific centres, and these youth may have been more comfortable sharing their orientation than others. Further, the lack of standardised measures for LGBT+ identity contributes to the variability of population estimates and can make comparisons difficult.

The majority of studies used cross‐sectional designs. Without detailed longitudinal studies and the control of confounding additional variables, it may be difficult to distinguish whether ACEs affect later outcomes or if participants recall more adversity when they have poor mental health outcomes. In cross‐sectional studies, participants may unintentionally underreport trauma if there are feelings of self‐blame, embarrassment, rumination biases or stigmatisation. Most studies were based in North America or in Europe, so data should be interpreted through a Western lens and not be overgeneralised to all cultures.

The meta‐analysis was also subject to limitation. We were unable to conduct a meta‐analysis using the subpopulations of LGBT+ as moderating factors since the data collected were not homogeneous. The sample sizes were not concordant, and the populations were broadly categorised, grouping together the small samples of those who did not identify as heterosexual. As a result, we note that our ability to speak to differences within sexual‐and gender minority populations is limited. Further to this, we were unable to distinguish between different gender identities (e.g., transgender man, transgender woman).

Most studies were based in the United States of America (US) or in Europe. Consequently, a generalisation of the data is limited to Western nations and can only be applicable to the rest of the world with caution. In such areas, an overwhelming majority hold the view that homosexuality or diverse gender identities is highly problematic and the consequences for expressing such an identity can be fatal. Although there are various reasons why it is difficult to obtain data from participants in these areas, future research should find creative ways to have a better understanding of ACEs and mental health outcomes in participants living within cultures under severe constraints and conflict zones to combat state‐sponsored repression and social stigma.

### Implications

#### How should schools prepare for working with LGBT+ youth?

The strong evidence that school‐based victimisation is causative of mental illness as well as suboptimal academic outcomes, less motivation and less engagement, highlights the need for establishing inclusive programming to support the mental health of LGBT+ youth.

It is well documented that the impact of childhood adversity on LGBT+ youth can be lessened through relationships with supportive peers. Theory supports that other peer‐based support groups would be helpful, and these have been piloted (e.g., Ramsey et al., 2015). Further, key adults must be vigilant in recognising victimisation efforts, strive to establish rapport with vulnerable students and offer a safe space where minority youth feel valued in their choices. For example, by always ensuring they are using a transgender youth's preferred pronouns.

#### How should clinicians prepare for working with LGBT+ youth?

Outside education institutions, continued advocacy is required to modify the longstanding history of poor patient care towards LGBT+ people (Riggs & Sion, 2017). Service professionals should aspire to foster inclusive and safe health care environments in which youth can disclose sensitive histories is recommended to avoid poor engagement and poor treatment adherence.

Clinicians assessing young people may wish to pay particular attention to patients endorsing a minority identity status and consider the aetiological role of trauma in their practice and adopt a trauma‐informed approach. This places a strong emphasis on enhancing support systems, mitigating trauma reminders and fostering safety. Alternatively, cognitive‐behavioural interventions may serve to alter negative self‐narratives attributed to minority stress in both sexual and gender minorities. Best practice may call for matching LGBT+ youth with clinicians where possible to provide positive role modelling or educate clinicians using evidence‐based care.

### Directions for future research

To enhance future study comparability and precision, a standardised sexuality/gender measure that possesses established validity may be of benefit. With only a small number of longitudinal studies in this literature base, future prospective studies would strengthen the hypothesis of causation. Given the disparities between LGBT+ youth and their peers, qualitative research can also shed more light on the lived experiences of marginalised community members. To help with disclosure and reduce nonresponse, researchers need to establish trust with the young person, providing clear professionalism and confidentiality.

Despite clear evidence that LGBT+ populations are among the most vulnerable groups for mental health problems, stigma has driven a long‐standing shortage of funding. It is important that LGBT+ specific work is continuously reviewed and updated in light of rapid developments in public discourse and academic knowledge.

## CONCLUSIONS

Existing literature has produced compelling evidence of the pervasive nature of the trauma that LGBT+ young people endure, but in the absence of systematic reviews, we offer an up‐to‐date understanding of the various sources of adversity which can contribute to poor mental health outcomes among LGBT+ youth. Information demonstrating higher prevalence of mental health disorders among LGBT+ individuals has been published previously, but the present results suggest that they emerge in adolescence. In addition to confirming and extending findings related to ACEs in LGBT+ individuals, we also documented an association between ACEs and poor mental health in transgender respondents. Continued advocacy is needed from communities and Allies to support and empower LGBT+ youth in the face of adversity. Longitudinal and longer‐term studies are required to further understand the relationship between adverse experiences in LGBT+ youth and the impact on mental health.

## CONFLICT OF INTEREST

The authors declare that they have no competing or potential conflicts of interest.

## ETHICS STATEMENT

No ethical approval was required for this review.

## AUTHOR CONTRIBUTIONS

All authors meet criteria for authorship and have approved the final version of the manuscript. LJ had full access to all of the data in the study and takes responsibility for the integrity of the data and the accuracy of the data analysis.

## Supporting information

Supporting Information S1

## Data Availability

The data that support the findings of this study are available from the corresponding author up‐on reasonable request.
