# Supplementary material for: A systematic review and meta‐analysis investigating the impact of childhood adversities on the mental health of LGBT+ youth
Source: JCPP Adv. 2022 May 10;2(2):e12079. doi: 10.1002/jcv2.12079 (PMC10242973; doi:10.1002/jcv2.12079)
Supplement: Supplementary file 1 — Supporting Information S1 [file JCV2-2-e12079-s001.docx]

**A Systematic Review and Meta-Analysis Investigating the Impact of Childhood Adversities on the Mental Health of LGBT+ Youth**

Supporting Information:

1. **Table S1**: Full Search Strategy
2. **Table S2**: PRISMA Checklist
3. **Table S3**: MOOSE Checklist
4. **Table S4:** Newcastle Ottawa Scale (Cross Sectional Studies and Longitudinal Studies)
5. **Table S5**: Studies included in Systematic Review (Country, Design & Recruitment)
6. **Table S6**: Breakdown of LGBT+ (%) Samples
7. **Table S7:** Sample Characteristics of Included Studies
8. **Table S8:** Risk of bias (quality assessment) using modified Newcastle Ottawa Scale for Cross Sectional Studies
9. **Table S9:** Risk of bias (quality assessment) using modified Newcastle Ottawa Scale for longitudinal studies.
10. **Supplementary information**: Further Meta-Analysis Information (Inc. Figure S1 and Figure S2).
11. **References**

This supporting information has been provided by the authors to give readers additional information about their work.

**Table S1: PRISMA Checklist (Page et al., 2021)**

| **Section and Topic** | **Item #** | **Checklist item** | **Location where item is reported** |
| --- | --- | --- | --- |
| **TITLE** | | |  |
| Title | 1 | Identify the report as a systematic review. | Title (pg. 1) |
| **ABSTRACT** | | |  |
| Abstract | 2 | See the PRISMA 2020 for Abstracts checklist. | Abstract (pg. 3) |
| **INTRODUCTION** | | |  |
| Rationale | 3 | Describe the rationale for the review in the context of existing knowledge. | Introduction (pg. 4) |
| Objectives | 4 | Provide an explicit statement of the objective(s) or question(s) the review addresses. | Introduction (pg. 4) |
| **METHODS** | | |  |
| Eligibility criteria | 5 | Specify the inclusion and exclusion criteria for the review and how studies were grouped for the syntheses. | Method (pg. 5) |
| Information sources | 6 | Specify all databases, registers, websites, organisations, reference lists and other sources searched or consulted to identify studies. Specify the date when each source was last searched or consulted. | Method (pg. 5) |
| Search strategy | 7 | Present the full search strategies for all databases, registers and websites, including any filters and limits used. | Method (pg. 5) |
| Selection process | 8 | Specify the methods used to decide whether a study met the inclusion criteria of the review, including how many reviewers screened each record and each report retrieved, whether they worked independently, and if applicable, details of automation tools used in the process. | Method (pg. 6) |
| Data collection process | 9 | Specify the methods used to collect data from reports, including how many reviewers collected data from each report, whether they worked independently, any processes for obtaining or confirming data from study investigators, and if applicable, details of automation tools used in the process. | Method (pg. 6) |
| Data items | 10a | List and define all outcomes for which data were sought. Specify whether all results that were compatible with each outcome domain in each study were sought (e.g. for all measures, time points, analyses), and if not, the methods used to decide which results to collect. | Method (pg. 6) |
|  | 10b | List and define all other variables for which data were sought (e.g. participant and intervention characteristics, funding sources). Describe any assumptions made about any missing or unclear information. | Method (pg. 6) |
| Study risk of bias assessment | 11 | Specify the methods used to assess risk of bias in the included studies, including details of the tool(s) used, how many reviewers assessed each study and whether they worked independently, and if applicable, details of automation tools used in the process. | Method (pg. 6) |
| Effect measures | 12 | Specify for each outcome the effect measure(s) (e.g. risk ratio, mean difference) used in the synthesis or presentation of results. | Method (pg. 6 & 7) |
| Synthesis methods | 13a | Describe the processes used to decide which studies were eligible for each synthesis (e.g. tabulating the study intervention characteristics and comparing against the planned groups for each synthesis (item #5)). | Method (pg. 7) |
|  | 13b | Describe any methods required to prepare the data for presentation or synthesis, such as handling of missing summary statistics, or data conversions. | Method (pg. 7) |
|  | 13c | Describe any methods used to tabulate or visually display results of individual studies and syntheses. | Method (pg. 7) |
|  | 13d | Describe any methods used to synthesize results and provide a rationale for the choice(s). If meta-analysis was performed, describe the model(s), method(s) to identify the presence and extent of statistical heterogeneity, and software package(s) used. | Method (pg. 7) |
|  | 13e | Describe any methods used to explore possible causes of heterogeneity among study results (e.g. subgroup analysis, meta-regression). | Method (pg. 7) |
|  | 13f | Describe any sensitivity analyses conducted to assess robustness of the synthesized results. | Method (pg. 7) |
| Reporting bias assessment | 14 | Describe any methods used to assess risk of bias due to missing results in a synthesis (arising from reporting biases). | Method (pg. 6) |
| Certainty assessment | 15 | Describe any methods used to assess certainty (or confidence) in the body of evidence for an outcome. | Method (pg. 7) |
| **RESULTS** | | |  |
| Study selection | 16a | Describe the results of the search and selection process, from the number of records identified in the search to the number of studies included in the review, ideally using a flow diagram. | Results (pg. 8) |
|  | 16b | Cite studies that might appear to meet the inclusion criteria, but which were excluded, and explain why they were excluded. | Results (PRISMA flow diagram) |
| Study characteristics | 17 | Cite each included study and present its characteristics. | Results (eTable X)). |
| Risk of bias in studies | 18 | Present assessments of risk of bias for each included study. | Results (eTable 9) |
| Results of individual studies | 19 | For all outcomes, present, for each study: (a) summary statistics for each group (where appropriate) and (b) an effect estimate and its precision (e.g. confidence/credible interval), ideally using structured tables or plots. | Results |
| Results of syntheses | 20a | For each synthesis, briefly summarise the characteristics and risk of bias among contributing studies. | Results |
|  | 20b | Present results of all statistical syntheses conducted. If meta-analysis was done, present for each the summary estimate and its precision (e.g. confidence/credible interval) and measures of statistical heterogeneity. If comparing groups, describe the direction of the effect. | Results (pg. 11, 12 and eFigure X) |
|  | 20c | Present results of all investigations of possible causes of heterogeneity among study results. | Results (pg 11) |
|  | 20d | Present results of all sensitivity analyses conducted to assess the robustness of the synthesized results. | Results |
| Reporting biases | 21 | Present assessments of risk of bias due to missing results (arising from reporting biases) for each synthesis assessed. | Results |
| Certainty of evidence | 22 | Present assessments of certainty (or confidence) in the body of evidence for each outcome assessed. | Results |
| **DISCUSSION** | | |  |
| Discussion | 23a | Provide a general interpretation of the results in the context of other evidence. | Discussion (pg. 13) |
|  | 23b | Discuss any limitations of the evidence included in the review. | Discussion (pg. 15) |
|  | 23c | Discuss any limitations of the review processes used. | Discussion (pg. 15) |
|  | 23d | Discuss implications of the results for practice, policy, and future research. | Discussion (Supplementary information). |
| **OTHER INFORMATION** | | |  |
| Registration and protocol | 24a | Provide registration information for the review, including register name and registration number, or state that the review was not registered. | Method (pg. 5) |
|  | 24b | Indicate where the review protocol can be accessed, or state that a protocol was not prepared. | PROSPERO (pg. 5) |
|  | 24c | Describe and explain any amendments to information provided at registration or in the protocol. | N/a |
| Support | 25 | Describe sources of financial or non-financial support for the review, and the role of the funders or sponsors in the review. | Title page (pg. 1) |
| Competing interests | 26 | Declare any competing interests of review authors. | Title page (pg. 1) |
| Availability of data, code and other materials | 27 | Report which of the following are publicly available and where they can be found: template data collection forms; data extracted from included studies; data used for all analyses; analytic code; any other materials used in the review. |  |

| Criteria  **Table S2: MOOSE Checklist (Stroup et al., 2000)** | | Brief description of how the criteria were handled in the meta-analysis |
| --- | --- | --- |
| Reporting of background should include | |  |
| √ | Problem definition | No meta-analysis has evaluated the presence of a range of adverse experiences and mental health outcomes in LGBT+ young people. |
|  | Hypothesis statement | Addressed in the introduction |
| √ | Description of study outcomes | Study objectives were described in the introduction |
|  | Type of exposure or intervention used | Adverse childhood experiences were the exposure of interest |
| √ | Type of study designs used | Both cross sectional and longitudinal studies were selected |
| √ | Study population | Young people who identify as LGBT+ |
| Reporting of search strategy should include | |  |
| √ | Qualifications of searchers | The credentials of the investigators are indicated in the author list and in the acknowledgements |
| √ | Search strategy, including time period included in the synthesis and keywords | We performed a multi-step literature search using keywords described in methods section: until 1^st^ September |
| √ | Databases and registries searched | Web of Science database (Web of Science Core Collection, BIOSIS Citation Index, KCI-Korean Journal Database, MEDLINE, Russian Science Citation Index, and SciELO Citation Index) and grey literature |
| √ | Use of hand searching | References of systematic reviews or meta-analyses that were screened during literature search and the references from the included studies were manually searched |
| √ | List of citations located and those excluded, including justifications | Details of the literature search process are outlined in the results section and PRISMA flowchart |
| √ | Method of addressing articles published in languages other than English | Only articles in English language were selected |
| √ | Method of handling abstracts and unpublished studies | Original individual studies were included. Reviews, clinical cases and study protocols were excluded |
| √ | Description of any contact with authors | We did not contact authors |
| Reporting of methods should include | |  |
| √ | Description of relevance or appropriateness of studies assembled for assessing the hypothesis  to be tested | Detailed inclusion and exclusion criteria are described in the methods section |
| √ | Rationale for the selection and coding of data | Data extracted from each of the studies are relevant to the population characteristics, study design and studies outcomes |
| √ | Assessment of confounding | We did not investigate confounding factors as stated in the limitations section |
| √ | Assessment of study quality, including blinding of quality assessors; stratification or regression on possible predictors of study results | We evaluated the quality using Mixed Methods Appraisal tool |
| √ | Assessment of heterogeneity | Heterogeneity was assessed with the I^2^ index |
| √ | Description of statistical methods in sufficient detail to be replicated | A random-effects meta-analysis was used. Heterogeneity among study point estimates was assessed using Q statistics. The proportion of the total variability in the effect size estimates was evaluated with the I^2^ index |
| √ | Provision of appropriate tables and graphics | We included the PRISMA flow-chart to describe the literature search and its results |
| Reporting of results should include | |  |
| √ | Table summarizing individual study estimates and overall estimate | We reported this in the results |
| √ | Table giving descriptive information for each study included | We have presented descriptive information for each study in the tables and as supplementary material |
| √ | Results of sensitivity testing | We did not conduct subgroup analyses |
| √ | Indication of statistical uncertainty of findings | We reported this in the results section |
| Reporting of discussion should include | |  |
| √ | Quantitative assessment of bias | Publication biases was assessed using a modified version of the Newcastle-Ottawa Scale was used for cross-sectional and longitudinal studies. Scores ranged from 0 to 8 |
| √ | Justification for exclusion | We excluded studies based on the rationale of the meta-analysis |
| √ | Assessment of quality of included studies | The quality of the studies was assessed and reported |
| Reporting of conclusions should include | |  |
| √ | Consideration of alternative explanations for observed results | We have addressed this point in the discussion section |
| √ | Generalization of the conclusions | We have addressed this point in the discussion section |
| √ | Guidelines for future research | We have addressed this point in the discussion section |
| √ | Disclosure of funding source | We had no funding source |

**Table S3: Full Search Strategy**

| *Population* | 1. ‘LGBTQ+ Youth’ 2. Lesbian 3. Gay 4. Homosexual 5. Same sex 6. Bisexual 7. ‘Sexual Minorit*’ 8. Transgender 9. ‘Gender diverse’ 10. ‘Gender nonconforming’ 11. ‘Non-binary’ 12. ‘Transgender young people’ 13. ‘Transgender girls’ 14. ‘Transgender boys’ 15. Transexual 16. Intersex 17. Pansexual 18. Asexual 19. Queer 20. OR/1-20 |
| --- | --- |
| *Exposure* | 1. Child* adverse experience 2. Child* sexual abuse 3. Child* emotional abuse 4. Child* physical abuse 5. Child* neglect 6. Traumatic experiences 7. Adverse events 8. Stressful events 9. ‘Household dysfunction’ 10. ‘Bullying’ 11. ‘Cyberbullying’ 12. Discrimination 13. ‘Openly gay’ 14. ‘Openly transgender’ 15. Violen* 16. OR/22-39 |
| *Outcome* | 1. Mental health 2. Mental disorders 3. Psychiatric 4. Emotional dysregulation 5. Mood disorders 6. Emotional disorders 7. Behavioural disorders 8. Eating disorders 9. Substance abuse 10. Post-traumatic stress disorder 11. Emotional Unstable Personality Disorder 12. Attachment Disorders 13. Neurodevelopmental conditions 14. Learning disabilities 15. Soiling 16. Enuresis 17. OR/41-58 |

**Table S4: Newcastle-Ottawa Quality Assessment for Cross sectional Studies and Longitudinal studies** (Alameda et al., 2020; Modesti et al., 2016)

| Criteria | Maximum Score |
| --- | --- |
| *Cross-Sectional Studies* | |
| Sample representative of target sample (e.g., all eligible or random sample)? | 1 |
| Sample size justified and satisfactory? | 1 |
| Non-response rate is defined, satisfactory, and characteristics of responders/non-responders compared? | 1 |
| Ascertainment of exposure is valid and/or well-described? | 2 |
| The subjects in different outcome groups are comparable and confounding are controlled? | 2 |
| Assessment of outcome with robust tool and/or record linkage? | 1 |
| Outcome per group reported appropriately? | 1 |
| *Longitudinal Studies* | |
| Representativeness of exposed cohort (e.g. total population or random sample, selected group) | 1 |
| Exposed and unexposed are matched or adjustment for confounding factors? | 1 |
| Method used to ascertain nonexposed cohort is robust? | 1 |
| Study controls for important and additional factors (e.g., LGBT+ status, demographics) | 1 |
| Outcome assessment was robust | 1 |
| Follow-up period was sufficiently long for outcomes to occur? | 1 |
| Loss to follow-up rate is reported, low (<80%), and same in exposed and non-exposed? | 1 |

**Table S5:** *Study characteristics* *of identified studies included in the systematic review (ordered alphabetically by first author)*

| **First Author** | **Year** | **Study Name** | **Country** | **Design** | **Recruitment** |
| --- | --- | --- | --- | --- | --- |
| Attebery-Ash | 2020 | Forced sex among youth: accrual of risk by gender identity, sexual orientation, mental health and bullying. | United States | Cross sectional | 2015 Healthy Kids Colorado Survey (HKCS) |
| Baams | 2018 | Minority Stress, perceived burdensomeness, and depressive symptoms among sexual minority youth. | Netherlands | Cross sectional | Advertisements on LGBT+ directed websites. |
| Battalen | 2020 | Associations of Discrimination, Suicide Ideation Severity and Attempts, and Depressive Symptoms Among Sexual and Gender Minority Youth | United States | Cross sectional | Community drop-in centres and other non-profit organizations providing services to LGBT+ youth in north-eastern United States. |
| Birkett | 2009 | LGB and Questioning Students in Schools: The Moderating Effects of Homophobic Bullying and School Climate on Negative Outcomes | United States | Longitudinal | The Dane County Youth Assessment (DCYA) is administered to 7–12th graders in 5-year intervals |
| Burton | 2014 | Sexual Minority-Related Victimization as a Mediator of Mental Health Disparities in Sexual Minority Youth: A Longitudinal Analysis | United States | Cross sectional | Youth were recruited to participate from a clinic in Pennsylvania or in Ohio by either provider referral or a screening system. |
| Button. | 2014 | Understanding the Effects of Victimization: Applying General Strain Theory to the Experiences of LGBQ Youth | United States | Cross sectional | Biannual survey in 2003 2005, and 2007 in public high schools in the state of Delaware. |
| Byrd. | 2015 | The Role of Cyberbullying Victimisation in Sexual Minority Adolescents' Reported Levels of Depression and Anxiety | United States | Cross sectional | Convenience sampling and snowball-sampling techniques from community organizations to obtain LGBT+ students a comparison group of heterosexual participants. |
| Chodzen | 2018 | Minority Stress Factors Associated with Depression and Anxiety Among Transgender and Gender-Nonconforming Youth | United States | Cross sectional | Adolescent patients presenting for care at a multidisciplinary gender clinic. |
| D’Augelli | 2006 | Childhood Gender Atypicality, Victimisation, and PTSD Among Lesbian, Gay and Bisexual Youth. | United States | Cross sectional | Attending community-based organisations in New York City and surrounding suburbs. |
| DeLaney | 2020 | The Associations Between Sexual Victimization and Health Outcomes Among LGBQA College Students: Examining the Moderating Role of Social Support | United States | Cross sectional | The students recruited in this current study were obtained from a large longitudinal study (Dick et al., 2014) |
| Donahue | 2017 | Familial Factors, Victimization, and Psychological Health Among Sexual Minority Adolescents in Sweden | Sweden | Cross sectional | The Child and Adolescent Twin Study (ongoing prospective, longitudinal study). |
| Duarte | 2017 | Correlation of Minority Status, Cyberbullying, and Mental Health: A Cross-Sectional Study of 1031 Adolescents | United States | Cross sectional | The data was obtained from an intervention study for high-risk adolescents. |
| Garaig-ordibil | 2020 | Bullying and cyberbullying in LGBT adolescents: Prevalence and effects on mental health | Spain | Cross sectional | A stratified sampling technique was used to represent the students of the last cycle of Secondary Education of the Basque Country |
| Hatchel | 2017 | Sexual Harassment Victimization, School Belonging, and Depressive Symptoms Among LGBTQ Adolescents: Temporal Insights | United States | Longitudinal | Students enrolled in six Midwestern public high schools were invited to complete in-school surveys in Spring 2014, 2015, 2016. |
| Huebner | 2015 | School Victimization and Substance Use among Lesbian, Gay, Bisexual, and Transgender Adolescents | United States | Cross sectional | Fliers, online social networking sites, direct outreach to youth who attended programming at the community-based organizations, and word-of-mouth. |
| Jones | 2017 | Anxiety Disorders, Gender Nonconformity, Bullying and Self Esteem in Sexual Minority Adolescents: Prospective cohort study | United Kingdom | Longitudinal | Secondary data was sourced from the Avon Longitudinal Study of Parents and Children (ALSPAC) |
| Kurki-Kangas | 2020 | Associations between Involvement in Bullying and Emotional and Behavioral Symptoms: Are there Differences between Heterosexual and Sexual Minority Youth? | Finland | Cross sectional | The survey is conducted among 8th and 9th graders of comprehensive school and 2nd year students of upper secondary and vocational school. |
| Li | 2019 | Research on Relationships between Sexual Identity, Adverse Childhood Experiences and Non-Suicidal Self-Injury among Rural High School Students in Less Developed Areas of China | China | Cross sectional | A high school in Jiangxi province was selected by the convenient sampling method. |
| Lowry | 2020 | Violence Victimization, Substance Use Disparities, and Gender-Nonconforming Youth | United States | Cross sectional | Questionnaires were administered in public high schools. |
| McNamee | 2008 | Same sex attraction, homophobic bullying and mental health of young people in Northern Ireland | Northern Ireland | Cross sectional | 2005 Young Life and Times Survey (YLT) via postal questionnaire |
| Mereish | 2017 | Sexual orientation, minority stress, social norms, and substance use among racially diverse adolescents | United States | Cross sectional | A probability sample of middle and high school students using random cluster methods. |
| Mittleman | 2019 | Sexual minority bullying and mental health from early childhood through adolescence | United States | Longitudinal | Analyses used data from the Fragile Families and Child Wellbeing Study, a population-based cohort study of children born in 20 American cities. |
| Peters | 2020 | Sexual orientation differences in non-suicidal self-injury, suicidality, and psychosocial factors among an inpatient psychiatric sample of adolescents | United States | Cross sectional | From an adolescent psychiatric inpatient unit, who were admitted due to concerns of suicide risk. |
| Pollit | 2017 | Disclosure Stress, Social Support, and Depressive Symptoms Among Cisgender Bisexual Youth | United States | Cross sectional | From community agencies, college groups for LGBTQ youth or referred by existing participants. |
| Price-Feeney | 2018 | The relationship between bias-based peer victimisation and depressive symptomatology across sexual and gender identity | United States | Cross sectional | Participants were recruited from the Harris Poll Online (HPOL) opt-in panel. For LGBT youth, ppts were recruited through referrals from GLSEN. |
| Veale | 2017 | Enacted Stigma, Mental Health, and Protective Factors Among Transgender Youth in Canada | Canada | Cross sectional | Study investigators’ networks, online advertisements, social media sites, and by the study’s researchers contacting Canadian LGBT+ community organisations. |
| Zhao | 2020 | Association Among Maltreatment, Bullying and Mental Health, Risk Behaviour and Sexual Attraction in Chinese Students. | China | Cross sectional | A multistage stratified cluster sampling method was employed in Guangdong Province in China. |
|  |  |  |  |  |  |

**Table S6.** *Breakdown of LGBT+ participants in selected study samples by percentage.*

| **Author** | **Opposite Sex Attracted (OSA)** | **Same Sex (SSA)** | **Both (BSA)** | **Other (i.e., Questioning/ Pansexual)** | **Transgender** | **Gender non-conforming** | **Non-binary** |
| --- | --- | --- | --- | --- | --- | --- | --- |
| Attebery Ash | 87.6 | 1.7 | 6.7 | 4 | 1.6 | - | - |
| Baams | - | 49 | 22.1 | 8.5 | - | - | - |
| Battalen | 5 | 34 | 27 | 7 | 16 | - | - |
| Birkett | 75.2 | 10.5 | | 4.6 | - | - | - |
| Burton | 69.5 | 29 | | - | - | - | - |
| Button | - | 20.2 | 58.5 | 21.30 | - | - | - |
| Byrd | 54.3 | 16.5 | 14.6 | 18.3 | 3.4 | - | - |
| Chodzen | - | - | - | - | 86.2 | 13.8 | - |
| D’Augelli | - | 100 | | | - | - | - |
| DeLaney | - | 24 | 44 | 32 | - | - | - |
| Donahue | - | 9.4 | | | - | - | - |
| Duarte | 92.6 | 7.4 | | | - | - | - |
| Garaigordibil | 87.5 | 12.5 | | | - | - | - |
| Hatchel |  | 18.8 | 65.4 | 15.8 | - | - | - |
| Huebner |  | 59.7 | 26.2 | 14.1 | - | - | - |
| Jones | 88.7 | 11.3 | | - | - | - | - |
| Kurki-Kangas | 88.9 | 11.09 | | | - | - | - |
| Li | 82.87 | 3.6 | | 7.4 | - | - | - |
| Lowry | - | - | - | - | - | 17 | - |
| McNamee | 91.2 | 8.8 | | - | - | - |  |
| Mereish | 78.2 | 1.1 | 3.4 | 17.3 | 0.9 | - | - |
| Mittleman | 89.01 | 10.9 | | - | - | - |  |
| Peters | 48 | 3.8 | 31 | 17.3 | 15 | | - |
| Pollit | - | - | 100 | - | - | - | - |
| Price-Feeney | 61.6 | 16.9 | 18.9 | 2.6 | 3.4 | 4.6 |  |
| Veale | - | - | - | - | 53.2 | - | 39.6 |
| Zhao | 91.7 | 1.3 | 7 | - | - | - | - |

*Note.* The rows which share percentages (% ) (e.g., between SSA*,* BSA and Other) dichotomised sexuality between heterosexual and non-heterosexual or sometimes other.

| **First Author**  **Table S7:** *Sample characteristics of identified studies included in the systematic review* | **Subpopulations** | **Sexuality/Gender Measure** | **Sample Size** | **Age (Mean, SD)** | **Female (%)** |
| --- | --- | --- | --- | --- | --- |
| Attebery Ash | LGBT+ | “Which of the following best describes you? Heterosexual (straight), gay or lesbian, bisexual, and not sure.” | 14, 129 | 15.7 (1.19) | 51.2 |
| Baams | LGBT+ | Participants' responses to the sexual attraction question ranged from only other-sex attractions to only same-sex attractions. | 267 | 17.61 (1.87) | 71.20 |
| Battalen | LGBT+ | Sexual orientation was categorized as heterosexual, lesbian/gay, bisexual, something else, or don’t know. Gender was categorized as female, male, trans-female, trans-male, genderqueer, or other. | 94 | 18 (2.88) | 36 |
| Birkett | LGB+ | ‘‘Do you ever feel confused about whether you are lesbian, gay, or bisexual?’’ | 7,376 | 12-14 | 50.7 |
| Burton | LGB+ | “Please choose the description that best fits how you think about yourself.” | 197 | 17 (1.36) | 70 |
| Button | LGB+ | The data from this project used adolescents who identified as sexual minorities from a previous project. | 484 | 15.94 | 69.7 |
| Byrd | LGBT+ | Participants were asked to provide their sexual orientation (gay, lesbian, bisexual, transgender, unsure/questioning, heterosexual/straight, and other) | 206 | 15-18 | 68.40 |
| Chodzen | TGNC | Demographic characteristics were collected by youth’s parents. | 109 | 15.46 (1.55) | 71.6 |
| D’Augelli | LGB+ | Youth were asked about the ages at which important milestones related to the development of their sexual orientation occurred. | 528 | 17.03 (1.27) | 48 |
| DeLaney | LGB+ | Self-report measure of sexual orientation. | 234 | 18.46 (0.41) | 74 |
| Donahue | LGB+ | Which do you think best describes your sexual orientation today? Options: homosexual, bisexual, heterosexual, and other. | 4898 | 18 (0.0) | 59.3 |
| Duarte | LGB+ | Validated questions on sexual orientation. | 1,031 | 14.9 (1.39) | 52.2 |
| Garaigordibil | LGBQ | sociodemographic questionnaire requesting information on sexual orientation. | 1,748 | 13-17 | 52.6 |
| Hatchel | LGBT+ | Participants were asked to report sexual orientation. | 404 | T1: 15.27 | 45.30 |
| Huebner | LGBT+ | Audio Computer Assisted Self-Interview (ACASI) system assessment: current gender - female, male or transgender; sexual orientation - gay/lesbian, bisexual, queer, other. | 504 | 17.4 (1.4) | 42.7 |
| Jones | LGBT+ | Participants were asked to choose from a list, ‘the description that best ﬁts how you think about yourself’. | 4563 | T1: 15.5  T2: 17.5 | 56.20 |
| **First Author** | **Subpopulations** | **Sexuality/Gender Measure** | **Sample Size** | **Age (Mean, SD)** | **Female (%)** |
| Kurki-Kangas. | LGB+ | “Have you had a crush on or been in love with…”, with response options girl(s)/boy(s)/both/no I haven’t/I don’t know. Those in upper (U) education were asked “Are you sexually interested in ...” with response alternatives. | 120,400 | 16.57 (1.23) | C: 50.1  U: 52.5 |
| Li | LGB+ | “Which of the following best describes you?” Responses included heterosexual, gay or lesbian, bisexual, and questioning (the participants were questioning about their sexual identity). | 1810 | 15-18 | 44.8 |
| Lowry | LGB+ | "How do you think people at school would describe you?” Response ranged from very feminine to very masculine." “What is your sex?” (Response options: female, male), a 7-point gender nonconformity scale was created. | 6,082 | Grades 9-12 | 47.90 |
| McNamee | LGB+ | A statement pertaining to feeling sexually attracted to (1) only females and never to males (2) more often to females and at least once to a male (3) about equally often to females and males (4) more often to males and at least once to a female (5) only to males and never to females (6) to no-one at all. | 868 | 16 (0.0) | 59.40 |
| Mereish | LGBT+ | Participants’ sexual orientation identity was assessed with one item: “How do you identify? | 3012 | 11-18 | 49.3 |
| Mittleman | LGB+ | “Have you ever liked a girl as more than just a friend?” and “Have you ever liked a boy as more than just a friend?” | 3,022 | T1: 5  T2: 9  T3: 15 | *n.r.* |
| Peters | LGB+ | Gender, sexual orientation, and race were collected via self-report | 52 | 15.6 (1.47) | 62 |
| Pollit | B only | "What is your birth sex?" "What is your gender identity?" and “How would you describe your sexual identity?” | 383 | 17.9 (0.4) | 66.50 |
| Price-Feeney | LGBT+ | *‘How would you describe your sexuality or sexual orientation?’* Response options: gay, lesbian, bisexual, straight/heterosexual, questioning, queer, other, and not sure. *What is your biological sex?” “What is your gender?”* | 5,542 | 15.70 (0.3) | 51.20 |
| Veale | TGNC | Self-report whether they identified as trans, genderqueer, or felt their gender did not match their body. | 323 | 14-18 | 11% (37% AFAB) |
| Zhao | LGB+ | Which gender do you think you are romantically attracted to?” and the options are “male,” “female,” “both male and female,” “neither male nor female,” “unsure,” and “unwilling to answer.” | 21,019 | 15 | 49 |

| **Table S8.** *Quality of assessment of included articles (adapted for cross-sectional studies)*  **Author (year)** | **Selection** | | | | **Comparability** | **Outcome** | | **Total** |
| --- | --- | --- | --- | --- | --- | --- | --- | --- |
|  | Representativeness of the sample | Sample size | Non respondents | Ascertainment of exposure | Comparability of cohorts. Confounders are controlled | Assessment of outcome | Statistical Test |  |
| Atteberry Ash | * | * | * | * | * | * | * | 8 |
| Baams | * | * | * | ** | ** | * | * | 9 |
| Battalen |  | * | * | ** | * | * | * | 8 |
| Birkett | * | * | * | ** | ** | * | * | 9 |
| Button | * | * | * | * | * | * | * | 7 |
| Byrd | * | * | * | ** | ** | * | * | 9 |
| Chodzen | * | * |  | ** |  | * | * | 6 |
| D’Augelli | * | * | * | ** | * | * | * | 8 |
| DeLaney | * | * |  | ** | ** | * | * | 8 |
| Donahue | * | * | * | * | ** | * | * | 8 |
| Duarte | * | * | * | * | ** | * | * | 9 |
| Garaigordibil | * | * | * | ** |  | * | * | 6 |
| Huebner | * | * | * | * | * | * | * | 7 |
| Kurki-Kangas | * | * | * | ** | * | * | * | 8 |
| Li | * | * | * | ** | ** | * | * | 9 |
| Lowry | * | * | * | * | ** | * | * | 9 |
| McNamee |  | * |  | ** | * | * | * | 5 |
| Mereish | * | * | * | * | ** | * | * | 8 |
| Peters |  |  | * | ** | * | * | * | 6 |
| Pollit | * | * | * | * | ** | * | * | 8 |
| Price-Feeney | * | * | * | * | ** | * | * | 8 |
| Veale | * | * | * | ** |  | * | * | 7 |
| Zhao | * | * | * | ** | * | * | * | 8 |

| **Author (year)** | **Selection** | | | | **Comparability** | **Outcome** | | | **Total** |  | |
| --- | --- | --- | --- | --- | --- | --- | --- | --- | --- | --- | --- |
|  | Representativeness of the exposed cohort | Selection of the non-exposed cohort | Ascertainment of exposure | Demonstration that outcome was not present at start of the study | Comparability of cohorts  based on the basis of design or analysis | Assessment of outcome | Was follow-up long  enough for outcomes to occur? | Adequacy of follow up of cohorts |  |  | |
| Burton et al. | * | * | * |  | ** |  | * | * | 7 |  | |
| Jones et al. | * | * | * |  | ** | * | * |  | 7 |  | |
| Mittleman, | * | * | * | * | ** |  |  | * | 7 |  | |
| Hatchel et al. | * | * | * | * | ** |  | * | * | 8 |  |  |

**Table S9.** *Quality of assessment of included articles (Adapted for longitudinal studies)*

**Supplementary information: Meta-Analysis Results**

***Proportion of LGBT+ youth exposed to any Adverse Childhood Experience****.* The meta-analytical results revealed that 55.2% (95%CI=37.8-71.3%, k=18, n=186,468) of LGBT+ youth were exposed to any adverse experience*.* (Figure 2). Heterogeneity across the studies was statistically signiﬁcant, (Q=30250.982, I2=99.984%, p<0.001). Egger's test result did reveal significant publication bias (t=2.294, p=0.036). The meta-funnel plot reporting publication bias is shown in eFigure 3.

*Proportion of LGBT+ youth exposed to Sexual Abuse.* Altogether, 6 independent studies reported on the presence of sexual abuse within an LGBT+ sample, showing 29.7% (95%CI=21.5-39.4%) of LGBT+ youth had suffered sexual abuse. Heterogeneity across the studies was statistically signiﬁcant, (Q=1052.267, I2=99.525, p<0.001).

*Proportion of LGBT+ youth exposed to Verbal Abuse.* In total, 7 studies informed on the presence of verbal abuse in LGBT+ youth. The meta-analytical result reporting verbal abuse in this sample was 28.7% (95%CI=18.3%-41.8%), with statistically significant heterogeneity (Q=1436.934, I2=00.582, p<0.001).

*Proportion of LGBT+ youth exposed to Physical Abuse.* A subset of studies (k=7) reported on physical abuse among LGBT+ youth. The meta-analytical result showed that 26.5% (95%CI=17.6-37.7%) of LGBT+ young people had experienced physical abuse. Heterogeneity between studies was statistically significant. (Q=1508.251, I2=99.602, p<0.001).

*Proportion of LGBT+ youth exposed to Cyberbullying.* The meta-analytical result of cyberbullying in LGBT+ youth showed that 19.1% (95%CI=10.4-32.5%, k=4) of the sample had undergone cyberbullying. Heterogeneity across the studies was statistically signiﬁcant, (Q=1375.616, I2=99.709, p<0.001).

Sensitivity analyses stratified by design, (cross-sectional vs. longitudinal studies), revealed that 56.1% (95%CI=37.0-73.6%, k=16) of LGBT+ individuals were exposed to any ACE at baseline, and 47.6% (95%CI=33.9-61.7%, k=2) of LGBT+ youth suffered any ACE during the follow-up period, showing not differences depending on the type of study (p=0.489).

***Proportion of LGBT+ youth with any Mental Disorder****:* the meta-analytical results revealed that 23.2% (95%CI=16.0-32.4%, k=8) of LGBT+ youth reported any mental health problem (Figure 3). Heterogeneity across the studies was statistically signiﬁcant (Q=1320.831, I2=99.470, p<0.001). Egger's test result did not reveal significant publication bias (Egger’s test t=0.893, p=0.406), evidenced by the meta-funnel plot that reports publication bias shown in eFigure 5.

*Meta-analysis of Depressive and Anxiety Symptoms in LGBT+ youth.* With the present database, a total of 8 samples were included in the meta-analytic estimation of LGBT+ individuals with depressive and anxiety symptoms. The formal meta-analysis revealed that depressive symptoms were present in 36.9% (95%CI=26.1-49.1%, k=5) of LGBT+ subjects. As per anxiety symptoms, the meta-analytical results revealed that anxiety was present in 31.5% (95%CI=25.6-38.2%, k=3) of LGBT+ youth. Heterogeneity was statistically signiﬁcant for both conditions (Q=2644.658, I2=99.849, p<0.001, and Q=367.5, I2=99.456, p<0.001 respectively).

Sensitivity analyses stratified by design, (cross-sectional vs. longitudinal studies), revealed that 24.3% (95%CI=12.9-40.9%, k=6) of individuals within the LGBT+ sample had any mental disorder at baseline, and 21.5% (95%CI=18.8-24.5%, k=2) of LGBT+ youth developed any mental health condition during the follow-up period, showing no differences depending on the type of study (p=0.696).

*Comparison between LGBT+ and Heterosexual youth and their relationship with Mental Disorders.*

*Association between LGBT+ vs Heterosexual with any Mental Disorder.* Association between LGBT+ vs Heterosexual with Anxiety. Considering the presence of anxiety symptoms in LGBT+, and compared to heterosexual individuals, the meta-analytic results of four studies showed a statistically significant association between belonging to the LGBT+ community and the presence of anxiety compared to heterosexual youth (OR=2.334, 95%CI=1.893-2.878, p<0.001). Heterogeneity across studies was statistically significant (Q=59.096, I2=96.616, p<0.001)

*Association between LGBT+ vs Heterosexual with Depression.* A subset of studies (k=4) allowed the analysis of the presence of any mental disorder within the LGBTQ+ sample compared to a heterosexual sample. Our analysis showed that any mental health problem is more frequent in LGBTQ+ youth, (OR=1.942, 95%CI=1.362-2.769), reaching statistical significance (p0.001). Heterogeneity between studies was statistically significant (Q=56.467, I2=94.687, p<0.001)

*Association between LGBT+ vs Heterosexual with Anxiety.* Considering the presence of anxiety symptoms in LGBT+, and compared to heterosexual individuals, the meta-analytic results of four studies showed a statistically significant association between belonging to the LGBT+ community and the presence of anxiety compared to heterosexual youth (OR=2.334, 95%CI=1.893-2.878, p<0.001). Heterogeneity across studies was statistically significant (Q=59.096, I2=96.616, p<0.001).

***Figure S1.*** *Proportion of LGBT+ youth exposed to any Adverse Childhood Experience. Publication bias - Funnel Plot.*

***Figure S2.*** *Proportion of LGBT+ youth with any Mental Disorder. Publication bias- Funnel Plot.*

**References**

Atteberry-Ash, B., Walls, N. E., Kattari, S. K., Peitzmeier, S. M., Kattari, L., & Langenderfer-Magruder, L. (2020). Forced sex among youth: Accrual of risk by gender identity, sexual orientation, mental health and bullying. *Journal of LGBT Youth*, *17*(2), 193-213.

Baams, L., Dubas, J., Russell, S., Buikema, R., & van Aken, M. (2018). Minority stress, perceived burdensomeness, and depressive symptoms among sexual minority youth. *Journal Of Adolescence*, *66*, 9-18. <https://doi.org/10.1016/j.adolescence.2018>

.03.015

Riggs, D. W., & Sion, R. (2017). Gender differences in cisgender psychologists’ and trainees’ attitudes toward transgender people. *Psychology of Men & Masculinity, 18*(2), 187-190. <https://doi.org/10.1037/men0000047>

Birkett, M., Espelage, D., & Koenig, B. (2009). LGB and Questioning Students in Schools: The Moderating Effects of Homophobic Bullying and School Climate on Negative Outcomes. *Journal Of Youth And Adolescence*, *38*(7), 989-1000. <https://doi.org/10.1007/s10964-008-9389-1>

Button, D. (2016). Understanding the Effects of Victimization: Applying General Strain Theory to the Experiences of LGBQ Youth. *Deviant Behavior*, *37*(5), 537-556. https://doi.org/10.1080/01639625.2015.1060787

Byrd, Jessica Lauryn (2015). "The Role of Cyberbullying Victimization in Sexual Minority Adolescents' Reported Levels of Depression and Anxiety". Dissertations. Paper 11.

Chodzen, G., Hidalgo, M., Chen, D., & Garofalo, R. (2019). Minority Stress Factors Associated With Depression and Anxiety Among Transgender and Gender-Nonconforming Youth. *Journal Of Adolescent Health*, *64*(4), 467-471. https://doi.org/10.1016/j.jadohealth.2018.07.006

D’Augelli, A., Grossman, A., & Starks, M. (2006). Childhood Gender Atypicality, Victimization, and PTSD Among Lesbian, Gay, and Bisexual Youth. *Journal Of Interpersonal Violence*, *21*(11), 1462-1482. https://doi.org/10.1177/0886260506293482

Duarte, C., Pittman, S., Thorsen, M., Cunningham, R., & Ranney, M. (2018). Correlation of Minority Status, Cyberbullying, and Mental Health: A Cross-Sectional Study of 1031 Adolescents. *Journal Of Child & Adolescent Trauma*, *11*(1), 39-48. https://doi.org/10.1007/s40653-018-0201-4

Garaigordobil, M., & Larrain, E. (2020). Bullying and cyberbullying in LGBT adolescents: Prevalence and effects on mental health. *Comunicar*, *28*(62), 79-90. https://doi.org/10.3916/c62-2020-07

Hatchel, T., Espelage, D., & Huang, Y. (2018). Sexual harassment victimization, school belonging, and depressive symptoms among LGBTQ adolescents: Temporal insights. *American Journal Of Orthopsychiatry*, *88*(4), 422-430. https://doi.org/10.1037/ort0000279

Huebner, D., Thoma, B., & Neilands, T. (2014). School Victimization and Substance Use Among Lesbian, Gay, Bisexual, and Transgender Adolescents.*Prevention Science*, *16*(5), 734-743. https://doi.org/10.1007/s11121-014-0507-x

Jones, A., Robinson, E., Oginni, O., Rahman, Q., & Rimes, K. (2017). Anxiety disorders, gender nonconformity, bullying and self-esteem in sexual minority adolescents: prospective birth cohort study. *Journal Of Child Psychology And Psychiatry*, *58*(11), 1201-1209. https://doi.org/10.1111/jcpp.12757

Kurki-Kangas, L., Fröjd, S., Haravuori, H., Marttunen, M., & Kaltiala, R. (2019). Associations between Involvement in Bullying and Emotional and Behavioral Symptoms: Are there Differences between Heterosexual and Sexual Minority Youth?. *Journal Of School Violence*, *19*(3), 309-322. https://doi.org/10.1080/15388220.2019.1691009

Lowry, R., Johns, M., & Robin, L. (2020). Violence Victimization, Substance Use Disparities, and Gender-Nonconforming Youth. *American Journal Of Preventive Medicine*, *58*(5), e159-e169. https://doi.org/10.1016/j.amepre.2019.12.021

McNamee, H., Lloyd, K., & Schubotz, D. (2008). Same sex attraction, homophobic bullying and mental health of young people in Northern Ireland. *Journal Of Youth Studies*, *11*(1), 33-46. https://doi.org/10.1080/13676260701726222

Mereish, E., Goldbach, J., Burgess, C., & DiBello, A. (2017). Sexual orientation, minority stress, social norms, and substance use among racially diverse adolescents. *Drug And Alcohol Dependence*, *178*, 49-56. <https://doi.org/10.1016/j.drugalcdep.2017.04.013>

Mittleman, J. (2019). Sexual Minority Bullying and Mental Health From Early Childhood Through Adolescence. *Journal Of Adolescent Health*, *64*(2), 172-178. <https://doi.org/10.1016/j.jadohealth.2018.08.020>

Modesti, P., Reboldi, G., Cappuccio, F., Agyemang, C., Remuzzi, G., & Rapi, S. et al. (2016). Panethnic Differences in Blood Pressure in Europe: A Systematic Review and Meta- Analysis. *PLOS ONE*, *11*(1), e0147601. https://doi.org/10.1371/journal.pone.0147601

Ramsey, J., DiLalla, L., & McCrary, M. (2015). Cyber Victimization and Depressive Symptoms in Sexual Minority College Students. *Journal Of School Violence*, *15*(4), 483-502. <https://doi.org/10.1080/15388220.2015.1100116>

Page MJ, McKenzie JE, Bossuyt PM, Boutron I, Hoffmann TC, Mulrow CD, et al (2021). The PRISMA 2020 statement: an updated guideline for reporting systematic reviews. BMJ;372:n71. doi: 10.1136/bmj.n71.

Peters, J., Mereish, E., Krek, M., Chuong, A., Ranney, M., & Solomon, J. et al. (2020). Sexual orientation differences in non-suicidal self-injury, suicidality, and psychosocial factors among an inpatient psychiatric sample of adolescents. *Psychiatry Research*, *284*, 112664. <https://doi.org/10.1016/j.psychres.2019.112664>

Pollitt, A., Muraco, J., Grossman, A., & Russell, S. (2017). Disclosure Stress, Social Support, and Depressive Symptoms Among Cisgender Bisexual Youth. *Journal Of Marriage And Family*, *79*(5), 1278-1294. <https://doi.org/10.1111/jomf.12418>

Veale, J., Peter, T., Travers, R., & Saewyc, E. (2017). Enacted Stigma, Mental Health, and Protective Factors Among Transgender Youth in Canada. *Transgender Health*, *2*(1), 207-216. <https://doi.org/10.1089/trgh.2017.0031>

Zhao, M., Xiao, D., Wang, W., Wu, R., Zhang, W., Guo, L., & Lu, C. (2021). Association among maltreatment, bullying and mental health, risk behavior and sexual attraction in Chinese students. *Academic pediatrics*, *21*(5), 849-857. <https://doi.org/10.1016/j.acap.2020.11.024>
